# Outcomes for Congenital Diaphragmatic Hernia in Three Decades: A Report From a UK Surgical Centre

**DOI:** 10.1111/apa.70295

**Published:** 2025-09-02

**Authors:** Wan Teng Lee, Paul D. Losty

**Affiliations:** ^1^ Institute of Systems Molecular and Integrative Biology University of Liverpool Liverpool UK; ^2^ Department of Paediatric Surgery Alder Hey Children's Hospital Liverpool Liverpool UK; ^3^ Division of Paediatric Surgery Ramathibodi Hospital Mahidol University Bangkok Thailand

**Keywords:** congenital diaphragmatic hernia, multidisciplinary clinic, patch, primary repair, survivorship

## Abstract

**Aims:**

Congenital diaphragmatic hernia (CDH) is associated with lung hypoplasia, pulmonary hypertension and high mortality. Three decades experience from a UK centre is reported.

**Methods:**

Medical records of CDH newborns between February 1990 and November 2021 and attending a multidisciplinary clinic were examined. Survival and health outcomes are recorded.

**Results:**

Of 220 CDH newborns, left‐sided defects were 177 (80%) cases. Diaphragm patch was required in 91 (41%) patients and 42 (19%) additionally required abdominal wall prosthesis. Materials deployed included Gore‐Tex biological Surgisis patches all had early recurrences. Diaphragmatic patch was significantly associated with fundoplication (*p* 0.005). Overall survival rate was 85%—(90% survival primary defect vs. 79% patch) (*p* 0.035). Comparing decades, the post‐2010 CDH study cohort was a sicker patient group comprising more index cases with cardiac malformations (69% vs. 28%, *p* < 0.001) requiring greater cardiovascular inotrope support (61% vs. 25%, *p* < 0.001) and ECMO (15% vs. 5%, *p* 0.023).

**Conclusion:**

While a modestly excellent 85% survival rate is reported over three decades, CDH management at this UK university surgical centre witnessed a growing complexity of patients with mixed severity phenotype(s). Future challenges remain to be solved to improve survival for the most complex vulnerable patients.

**Summary**

- Congenital diaphragmatic hernia (CDH) survival is reflective of disease severity.
- 85% survival at this UK university surgical centre had a low rate (%) of recurrence, a requirement (%) for fundoplication, and risks (%) of adhesive intestinal obstruction requiring re‐laparotomy.
- This study provides a benchmark that may help define ‘best practice’ in seeking to promote centralisation of CDH care to high volume treatment centres.

**Abbreviations**
CDH: congenital diaphragmatic hernia
CMV: conventional mechanical ventilation
ECMO: extracorporeal membrane oxygenation
GERD: gastroesophageal reflux disease
HFOV: high frequency oscillatory ventilation
iNO: inhaled nitric oxide

## 1 Introduction

Congenital diaphragmatic hernia (CDH) management is currently the subject of great debate with health outcome(s) showing varying survival rates (%) across high versus low volume centres in several world nations [1, 2, 3, 4, 5, 6]. Parent advocacy groups, stakeholders and health care professionals increasingly call for improved targeted research priorities to be better defined, which may or may not impact or direct a political shift towards centralisation to high volume centres where survival for CDH may be influenced by skilled health care teams caring for the most complex ‘high risk’ patients [1, 2, 3, 4, 5, 6, 7]. Contributing factor(s) leading towards improved survival (%) in recent years notably have included—elective delivery of antenatally diagnosed CDH babies at specialist centres, permissive hypercapnia with ‘gentle ventilation’ strategies, better intensive cardio‐respiratory supportive care, timely use of extracorporeal membrane oxygenation (ECMO) and delayed operative repair following stabilisation of labile physiology [8, 9, 10]. Against this background of emerging evidence we herein now report some three decades of experience detailing surgical management and outcomes of CDH newborns admitted to a UK university paediatric surgical centre.

## 2 Methods

ALL CDH index cases at Alder Hey Children's Hospital Liverpool, UK between 1 February 1990 and 1 November 2021 are reported. Patients were identified from fetal medicine service clinic(s) and hospital medical case records which documented–(a) mode of birth delivery, (b) gestation age, (c) sex, (d) birth weight, (e) antenatal diagnosis, (f) ‘patient journey’/health care management including–(i) use of conventional mechanical ventilation (CMV), (ii) high frequency oscillatory ventilation (HFOV), (iii) inhaled nitric oxide (iNO)/pharmacological support and (iv) ECMO. Operative outcomes with respect to laterality/side of CDH defect including primary repair versus patch–(a surrogate marker of disease severity), age at repair (days), CDH survival rate (%) with and without associated anomalies including congenital heart disease (CHD), hospital discharge and/or death were accurately recorded. Patient survivorship/morbidity detailed varied outcome metrics which included (i) CDH recurrence (%), (ii) intestinal bowel obstruction (%) requiring laparotomy and (iii) those having fundoplication (%) linked to gastroesophageal reflux disease (GERD). All survivors were routinely reviewed with outpatient office follow‐up co‐ordinated through a dedicated multidisciplinary CDH clinic staffed by a consultant paediatric surgeon, respiratory physician with MDT team input and support from dieticians, nutritionists and other health care providers.

### 2.1 Statistical Analysis

All data were analysed using chi squared and Fisher's exact test. Continuous data with a non‐normal distribution were compared using Mann–Whitney *U* test, with SPSS version 27 software (IBM, Armonk, New York, USA). *p* < 0.05 was considered statistically significant.

## 3 Results

Over the course of three decades—220 newborns (142 male [64.5%] and 78 [35.5%] female)—underwent CDH repair at Alder Hey Children's Hospital, Liverpool, UK (Table 1).

**TABLE 1:** Comparative results—study time periods pre‐2010 versus post 2010 CDH Groups.

	Total (*n* = 220)	1990–2010 (*n* = 110)	Post 2010 (*n* = 110)	*p*
Gestational age (weeks)	38.5 (37–39)	39 (38–40)	38 (37–39)	0.374
Birth weight (g)	3200 (2800–3610)	3108 (2800–3590)	3290 (2805–3685)	0.686
Side (R:L)	38:177	19:88	18:89	0.598
Antenatal diagnosis	97 (43%)	41 (38%)	56 (58%)	0.004
Vaginal delivery	115 (65%)	70 (67%)	45 (61%)	0.006
Cardiac	93 (47%)	30 (28%)	63 (69%)	< 0.001
Syndromic	10 (5%)	5 (5%)	5 (5%)	< 0.001
HFOV	61 (32%)	24 (22%)	37 (44%)	0.002
ECMO	18 (9%)	5 (5%)	13 (15%)	0.023
Age at repair (d)	3.5 (2–7)	4 (2–8)	2.25 (3–6)	0.054
Survivor	187 (85%)	92 (84%)	95 (86%)	0.706

Case load activity at our UK university surgical centre steadily increased from each decade period to the next with regional and wider national referrals such that in the first two decades under review, that is, 1990–2010–we had 110 index CDH cases versus 110 patients later treated in the era 2010–2021, Figure 1. Left side Bochdalek defects were documented in 177 patients (80%) with 38 babies having right side CDH (17%).

**FIGURE 1:**
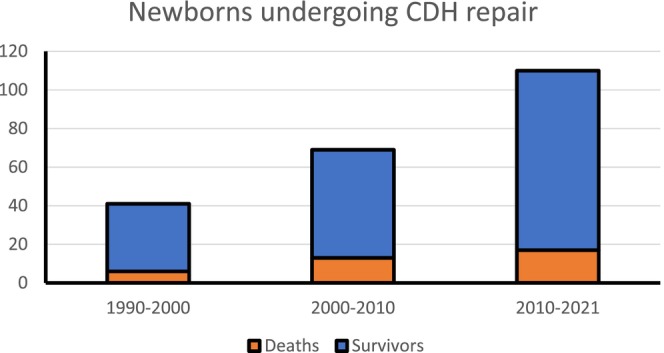
Time trends in operation repair rate(s) of congenital diaphragmatic hernia (CDH).

Vaginal birth delivery occurred in 115/220 (65%) of all index cases, with 56/110 (58%) of the CDH babies having an antenatal diagnosis recorded in the 2010–2021 time era(s)–(*p* 0.004). Gestational age and birth weights were noted to be similar when analysing all the index CDH cases across the pre and post 2010 time era periods–(*p* value = ns). CDH patients treated after 2010–2021 had notably much more severe complex disease phenotype(s) with a greater burden (%) of associated major anomalies, including cardiac malformations (*p* < 0.001) requiring HFOV support (*p* = 0.002) and ECMO (*p* = 0.023)—Tables. Analysis with respect to examining sex specific differences are listed in Table 2. We found overall rates (%) of cardiopulmonary intervention(s) support which included ECMO (*p* = 0.797) and survival (%) outcomes were similar comparing sexes (*p* = 0.846). Age and timing of operative CDH repair with improved and accumulating institutional expertise at physiological stabilisation of newborns progressively shortened the ‘time to operative surgery’ from some 4 days pre 2010 versus only 2.25 days in the 2010–2021 time era (*p* = 0.054). All CDH operations documented in this 30‐year study population were scheduled as ‘open classical repairs’ as at our institution we have previously reported × 3‐fold higher recurrence rate with minimally invasive surgery from a meta‐analysis study [11].

**TABLE 2:** Sex‐specific differences (%) in CDH patients.

	Total (*n* = 220)	Male (*n* = 142)	Female (*n* = 78)	*p*
Antenatal diagnosis	97 (43)	56 (40)	41 (53)	0.109
Cardiac	93 (47)	55 (38)	38 (49)	0.234
Syndromic	10 (5)	5 (4)	5 (6)	0.324
HFOV	61 (32)	37 (26)	24 (31)	0.518
ECMO	18 (9)	11 (8)	7 (9)	0.797
Diaphragmatic patch	91 (41)	54 (38)	37 (47)	0.148
Abdominal patch	42 (19)	29 (20)	13 (17)	0.717
Bowel obstruction	19 (12)	13 (9)	6 (8)	1.000
Recurrence	9 (6)	5 (4)	4 (5)	0.495
Fundoplication	14 (8)	6 (4)	8 (10)	0.146
Survivor	187 (85)	120 (85)	67 (86)	0.846

*Note:* Cardiac anomalies include‐major and minor congenital defects–ASD, VSD, Tetralogy of Fallot, aortic coarctation, hypoplastic arch disorders, valvular heart disease. Syndrome and chromosomal disorders included—Trisomy 21, Cornelia de Lange, Simpson‐Golabi‐Behmel syndrome, fetal alcohol syndrome, Kabuki syndrome, Pentalogy of Cantrell, chromosome 2 deletion, chromosome 3/chromosome 10 translocations, Glass syndrome, Cystic Fibrosis, Ehler Danlos syndrome, SATB2 associated syndrome(s).

Outcome metrics analysed which included—(i) CDH recurrence rate(s) (%), (ii) intestinal bowel obstruction(%) requiring laparotomy, (iii) antireflux surgery (%) notably Nissen fundoplication and (iv) mortality (%) trends pre 2010 and post 2010 years are detailed and listed in Tables 1, 2, 3, 4. We recorded a 6% CDH recurrence rate (%) during the 30 year study period under review. We routinely deployed Gore‐Tex (W.L. Gore & Associates, Arizona, USA) as prosthetic patch material of first choice. We have used biological patches—Surgisis–(Cook Biotech, Indiana, USA) however with two very early recurrences in newborns within weeks of CDH defect repair we no longer use biological patches. Intestinal obstruction requiring exploratory laparotomy with re‐operation was documented in 12% of patients. With multidisciplinary GI feeding management co‐ordinated though the CDH speciality clinic(s) only 8% total patients required a Nissen fundoplication for GERD.

**FIGURE 2:**
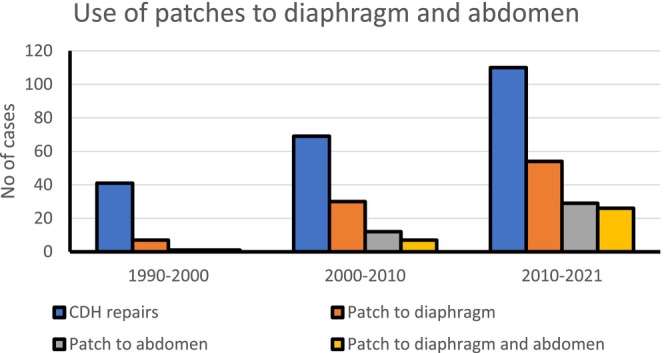
Trends in patch utilisation rate(s) in CDH study population.

Prosthetic patch utilisation for major sized CDH defect repair increased steadily over time, Figures 1 and 2, Tables 3 and 4. Examining trends diaphragm patches were deployed in < 20% index cases (1:5) during the 1990–2000 time year period. Diaphragm patch rates (%) progressively advanced to almost 50% index patients requiring CDH patch repair during the 2010–2021 era reflective of the greater severity CDH sized phenotype defects managed at our specialist centre. While higher diaphragmatic patch repair rates (47% vs. 38%) were recorded in female versus male patients, these findings, though of some interest to us, proved non‐significant (*p* = 0.148).

**TABLE 3:** CDH patient study population patch utilisation rate(s).

	1990–2000	2000–2010	Post‐2010	Total (%)
Patients with CDH	41	69	110	220
Patch to diaphragm	7 (17)	30 (43)	54 (49)	91 (41)
Patch to abdomen	1 (2)	12 (17)	29 (26)	42 (19)
Path to diaphragm and abdomen	1 (2)	7 (10)	26 (24)	35 (16)
Death	6 (12)	13 (19)	17 (15)	33 (15)

**TABLE 4:** Comparative results comparing CDH patch versus no patch study population.

	Total (*n* = 220)	Diaphragmatic patch (*n* = 91)	No patch (*n* = 129)	*p*
Gestational age (weeks)	38.5 (37–39)	38 (37–39)	39 (38–40)	< 0.001
Birth weight (g)	3200 (2800–3610)	2970 (2630–3420)	3275 (2955–3711)	0.003
Side (R:L)	38:177	23:66	14:111	0.003
Sex (M:F)	142:78	54:37	87:38	0.148
Antenatal diagnosis	97 (43%)	58 (72%)	39 (32%)	< 0.001
Vaginal delivery	115 (65%)	48 (63%)	66 (65%)	0.107
Cardiac	93 (47%)	49 (60%)	44 (39%)	0.004
Syndromic	10 (5%)	5 (6%)	5 (4%)	0.743
HFOV	61 (32%)	41 (51%)	19 (17%)	< 0.001
ECMO	18 (9%)	15 (19%)	3 (3%)	< 0.001
Age at repair (d)	3.5 (2–7)	5 (3–10)	3 (2–5)	0.135
Bowel obstruction	19 (12%)	13 (15%)	6 (9%)	0.326
Recurrence	9 (6%)	8 (9%)	1 (1%)	0.078
Fundoplication	14 (8%)	12 (14%)	2 (2%)	0.005
Survivor	187 (85%)	72 (79%)	112 (90%)	0.035

To avert abdominal compartment syndrome (ACS) developing in CDH patients, particularly in those ‘lacking sufficient abdominal domain’ abdominal wall patch use (%) has steadily grown in our CDH patient population, Figure 2, Table 3. Increasing deployment of abdominal wall patch prosthesis, together with patching the diaphragm, also emerged in the most recent study years to effect secure safe closure of the abdomen in those babies with the severest grade large size CDH defects. Mortality was notably significantly higher in babies requiring abdominal wall patch (*p* = 0.003), Table 3. With almost 50% of patients having prosthetic diaphragm repairs during 2010–2021, some 26% of CDH patients had abdominal wall patching alone, with 24% of the total index case load having synchronous abdominal wall and diaphragm prosthetic closure(s).

**FIGURE 3:**
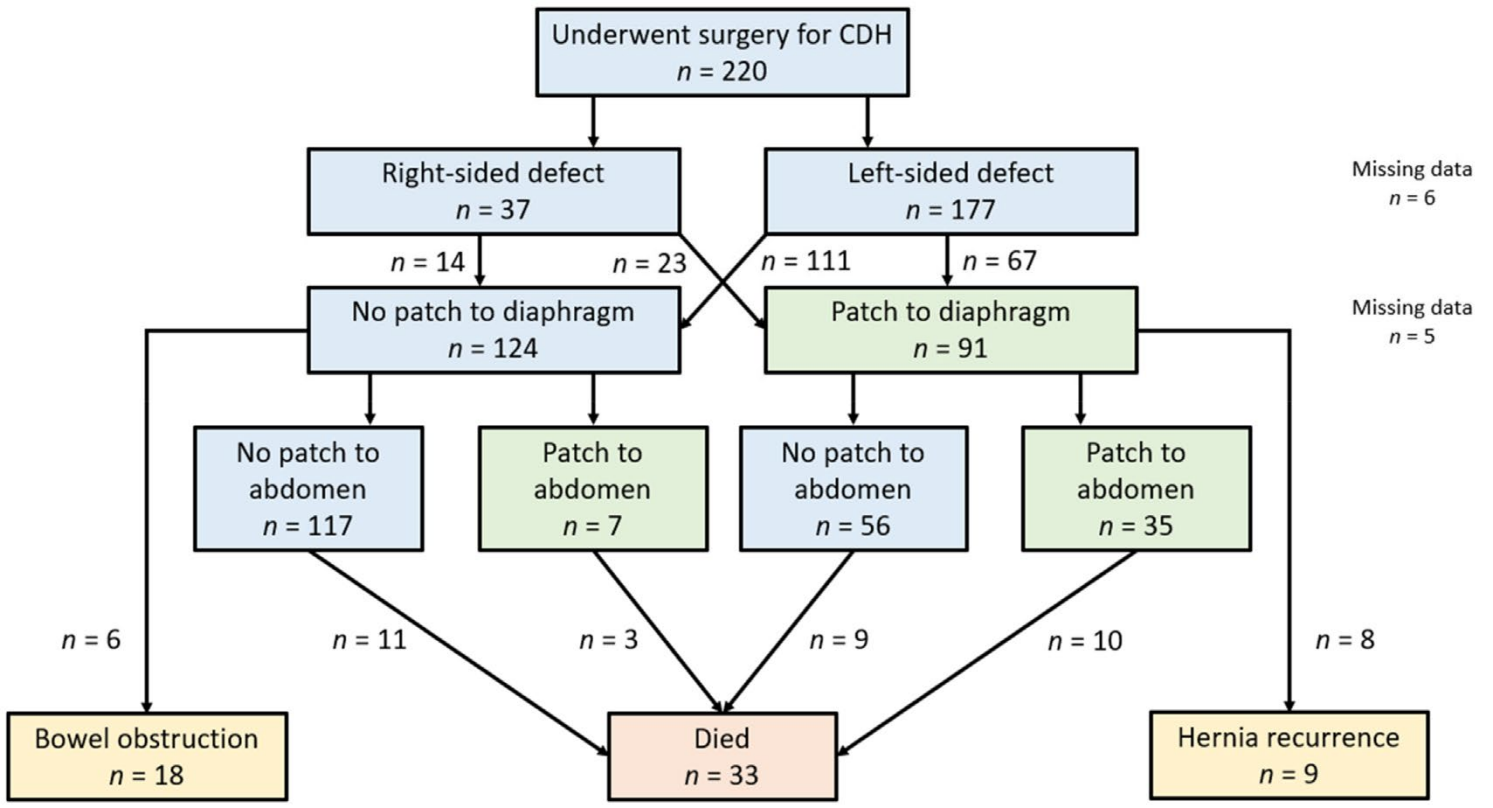
Summary flow chart depicting CDH outcomes reporting over three decades 1990–2021.

The flow diagram provides an overview summary chart, Figure 3. While right sided CDH was a less common defect phenotype, these patients had a higher patch rate requirement—24/37 (65%) versus left CDH 67/177 (38%). Analysing CDH patients that required diaphragm patching versus no patch these newborns were notably of a younger gestational age at delivery (*p* < 0.001) and had significantly lower birth weights (*p* = 0.003).

A higher rate of CDH detection antenatally (72% vs. 32%; *p* < 0.001) was recorded in babies requiring diaphragm patch linked to their severity size of defect, Table 3. The requirement for a diaphragm patch versus a primary repair operation was noted to be significantly higher also in CDH newborns with greater severity disease phenotypes; that is, in those who had specifically associated cardiac malformations (60% vs. 39%; *p* 0.004) showed increased requirement(s) for HFOV (51% vs. 17%; *p* < 0.001) and ECMO (19% vs. 3%; *p* = 0.001). Patch repair of the diaphragm did not significantly increase the later risk or rates (%) of intestinal obstruction (*p* = 0.326) or CDH recurrence (*p* = 0.078). The need for a fundoplication operation to treat GERD was greater in patients having CDH patch repair versus no patch (*p* = 0.005). Analysing female versus male CDH patients, notably 10% versus 4% (F: M) had a fundoplication for GERD (*p* = 0.146 NS). Of 187/220 index patients—an 85% CDH overall survival rate (%) is recorded. Survival (%) was significantly better in those having primary repair and no patch (90% vs. 79%, *p* = 0.035), Figure 1, Table 1.

## 4 Discussion

This study records a 30‐year experience detailing surgical management and outcomes of CDH at a UK university surgical centre. We report an 85% overall survival rate to primary hospital discharge at our institution caring for some of the most complex CDH patient phenotypes, including those with cardiac malformations. Newborns having primary native diaphragmatic hernia repair(s) had a 90% versus 79% survival rate documented in those patients considered a much ‘higher risk’ group with prosthetic patch repairs—(*p* = 0.035). These modestly excellent survival outcomes have been achieved at our UK centre, as in other institution reports, with improved antenatal care practice(s) including better perinatal management, implementing modern respiratory care strategies with permissive hypercapnia, HFOV, iNO use, pharmacological support methods, ECMO and delayed operative repair [8, 9, 10].

Antenatal CDH diagnosis improved significantly particularly during the latter 2010–2021 time era with a 58% anomaly detection rate, Table 1. These observations are broadly consistent with reports from the BAPS CASS CDH UK nationwide study and data emerging from European nation studies with a reported anomaly detection rate of some 60% [12, 13]. Consistent to previous study population based reports males (64.5%) had a higher incidence (%) of CDH versus females (35.5%) in this UK single centre study. Comparing the CDH patient groups according to sex, higher rates (%) of diaphragm patch repair were recorded in female versus males, which correlated with larger size phenotype defects [12, 14]. These findings sharply contrast with those published by the CDH Study Group which reported equivalent CDH sized defects and rates (%) of diaphragm patch repair among the sexes [15]. Mortality and morbidity rates were also strikingly similar between male and females in our UK study which again contrast with those from the CDH Study Group which showed female CDH patients were at much higher risk of mortality (%) and had a risk susceptibility to develop pulmonary hypertension [15].

ECMO utilisation was deployed in 18/220 (9%) CDH patients treated at Alder Hey Children's Hospital Liverpool, which is a considerably lower rate (%) of use of ECMO than that reported from published series emerging from North America [12]. Our single centre data of interest strikingly closely mirror and match some of the key working findings of the 2018 CDH BAPS CASS UK nationwide population‐based study, which again showed a comparatively low rate (%) of use of ECMO (4%) in an equivalent‐sized cohort of some 219 CDH patients treated across 28 paediatric surgical centres in Britain and Ireland [12]. Why these differences in ECMO utilisation rate(s) exist across world continents managing CDH deserves detailed scrutiny, investigation and future collaborative network studies.

From analysing surgical outcome metrics, it can be observed that we had a very low rate of CDH recurrence (6%) reflective we believe of the appropriate case selection of index patients for primary native diaphragm repair vs. those requiring prosthetic patches with largest sized CDH defects [16, 17]. For prosthetic patch insertion, we report a good institutional experience with Gore‐Tex (W.L. Gore and Associates, Arizona, USA) as the prosthetic patch material of our 1st choice [16, 17]. We have not witnessed the high rate(s) of patch disruption and CDH recurrence(s) as reported by others [18]. We would caution strongly, therefore, against the use of biological patches, where we had experienced two early recurrences within only weeks of primary operations [16, 19].

CDH patients requiring abdominal wall patching or ‘double patching’ of the diaphragm with abdominal wall prostheses in this current UK study were also reflective of the severity of disease phenotype [14, 16]. Few patients (8%) later required fundoplication for GERD, and these were notably often those CDH babies who had patch repairs (14%) with the largest sized diaphragm defects versus those newborns having primary CDH repair (2%)—*p* = 0.005, (Table 3) [16, 20].

We recorded a relatively low incidence rate (12%) of CDH survivors in this current study developing intestinal obstruction later then requiring a laparotomy with all index patients having had ‘classical open operations’ for CDH and remarkably also no increased risk burden (%) even in those having had CDH patch repairs versus primary native diaphragm repair (*p* = 0.326) [16, 21].

A multidisciplinary led CDH clinic—(1st UK CDH MDT clinic was established in Alder Hey Liverpool England)—modelled on an outpatient clinic facility inaugurated and originally developed by Jay M. Wilson MD at Boston Children's Hospital, USA—facilitated the dedicated and vigilant aftercare follow‐up of all patients and families allowing the health outcomes reporting in this study (CDH recurrence (%), fundoplication (%), adhesive intestinal obstruction (%)) [16, 22, 23]. From primary hospital discharge CDH patients are regularly monitored at monthly office visits—according to individual health needs—with gastrointestinal (GERD) and cardiorespiratory health surveillance (pulmonary function testing, echocardiography, CDH—chest x‐ray surveillance/recurrence) and transitional care then later co‐ordinated onwards to adult services for lifelong follow‐up where it is considered appropriate for each particular patient [24, 25, 26, 27]. Other health service providers, for example, surgical orthopaedics are involved with our CDH survivor study population as requested for scoliosis management (bracing or operative correction). Future ongoing studies are planned and will seek to later report longer‐term health outcomes.

An increasing number of studies show that high volume treatment centres and a shift towards centralisation may be linked to significantly better survival outcomes for CDH [1, 2, 3, 4, 5, 6]. A BAPS CASS UK nationwide study report in 2018 highlighted evidence of marked variation(s) in prenatal and postnatal management of CDH across hospital centres in the UK and Ireland, including deviation from ‘best practice’ likely impacting health outcomes [12]. MBRRACE (2014)—a confidential published enquiry—conducted across the UK into CDH care cited that improvements needed to be made to offset the disparities towards achieving better health outcomes in CDH [28]. We acknowledge despite the modestly excellent outcomes achieved at our UK centre that there are future challenges to be solved to improve outcomes further for the most complex vulnerable CDH patients.

In closing with some final remarks—We hope this current study highlighting three decades of experience at a UK university centre managing CDH may yet serve as another report and benchmark to drive progress towards centralisation of CDH care for index patients and affected families across world health care nations.

## Ethics Statement

The authors determined that this study did not require ethical approval by use of the NHS online tool.

## Conflicts of Interest

The authors declare no conflicts of interest.

## Data Availability

The data that support the findings of this study are available from the corresponding author upon reasonable request.
